# Factors influencing mental health service delivery during public health emergencies: a scoping review protocol

**DOI:** 10.12688/hrbopenres.13850.1

**Published:** 2024-02-16

**Authors:** Pawel Hursztyn, Almas Khan, Karen Matvienko-Sikar, Kairi Kõlves, Marguerite Nyhan, John Browne

**Affiliations:** 1 National Suicide Research Foundation, Cork, County Cork, Ireland; 2 School of Public Health, University College Cork, Cork, County Cork, Ireland; 3 Australian Institute for Suicide Research and Prevention, WHO Collaborating Centre for Research and Training in Suicide Prevention, School of Applied Psychology, Griffith University, Nathan, Queensland, Australia; 4 Discipline of Civil, Structural & Environmental Engineering, School of Engineering & Architecture, University College Cork, Cork, Ireland; 5 MaREI, The SFI Research Centre for Energy, Climate & Marine, University College Cork, Ringaskiddy, Cork, P43 C573, Ireland; 6 Environmental Research Institute, University College Cork, Cork, T23 XE10, Ireland

**Keywords:** Public Health Emergencies, Mental Health Interventions/Supports, Service Reconfiguration, Description, Effectiveness, Evaluation

## Abstract

**Background:**

Unforeseeable public health emergencies (PHEs) profoundly impact psychological well-being and disrupt mental health care provision in affected regions. To enhance preparedness for future emergencies, it is crucial to understand the effectiveness of mental health services, their underlying mechanisms, the populations they are tailored to, and their appropriateness across distinct emergencies. The aim of this scoping review will be to explore how mental health services have responded to PHEs, focusing on their effectiveness as well as barriers and facilitators to implementation.

**Methods:**

Following the five-stage Arksey-O'Malley guidance, as updated further by Westphaln and colleagues, this mixed-methods scoping review will search academic and grey literature. Publications related to mental health interventions and supports delivered during PHEs will be considered for inclusion. The interventions and supports are operationally defined as any adaptations to mental health service provision at the international, national, regional or community level as a consequence of PHEs. The “Four Ss” framework will be utilised to provide structure for the evidence synthesis and inform categorisation of interventions and supports delivered during PHEs. Any research methodology will be considered for inclusion. Two reviewers will independently screen titles, abstracts, and full texts of publications against eligibility criteria. The gathered data will be depicted in accordance with the Four Ss” framework through the utilisation of descriptive/analytical statistics and supplemented by narrative exploration of findings.

**Conclusions:**

Considering the diverse research methodologies and the varied applicability of services in different contexts of PHEs, this review will offer insights into the type, effectiveness, and implementation barriers and facilitators of mental health interventions and supports delivered during PHEs. By employing the “Four Ss” framework, the review will guide decision-making bodies in identifying effective and practical aspects of mental health system operations during emergencies.

## Background

Public health emergencies (PHEs) defined as “serious, sudden unexpected or unusual events that constitute a public health risk” ^1^, can be caused by disease outbreaks (e.g., pandemics, epidemics, local outbreaks), man-made or natural disasters (e.g., floods, hurricanes, earth quakes, bush fires), and war or military conflicts. PHEs can have a significant impact on the delivery of care for patients with pre-existing mental health conditions. For example, in the recent COVID-19 pandemic mental health services in many countries pivoted to remote out-patient care for their existing patient populations ^2^. The extent to which these new treatment modalities were successfully implemented and effective is the subject of ongoing research. A further PHE challenge for mental health care service providers is the creation of additional demand due to the increased incidence and prevalence of mental health conditions, e.g., severe anxiety, post-traumatic stress disorder (PTSD), depression, and acute stress disorders, as an immediate consequence of emergencies ^3– 5^.

While there is considerable evidence on the responses of mental health services to public health emergencies, the nature of this research varies not only methodologically (e.g., descriptive studies, evaluations highlighting barriers and facilitators of implementation, and effectiveness studies, in qualitative and quantitative forms) but also in the context of specific PHE type. For instance, a considerable body of evidence has investigated the effectiveness of various psychological interventions aimed at supporting the mental health of individuals who have been exposed to infectious disease outbreaks ^6^. Additionally, research has explored the effectiveness of tele-mental health and technology mediated interventions ^7, 8^. Fewer studies however, focused on the acceptability and usability of these mental health interventions ^9^.

The body of research pertaining to factors that facilitate or constrain the implementation of mental health interventions and supports subsequent to PHE is another recognisable domain. These factors encompass the adaptability of intervention to the emergency context, the unique characteristics of a region’s mental health system, and the specific needs of the affected individuals. They include a wide range of factors, including but not limited to the mental health system, mental health policies, financing, human and infrastructure resources, safety measures, privacy and confidentiality protocols, cultural considerations, and the impact of stigma ^10– 12^. Importantly, these factors remain similar in any type of emergency ^10– 12^.

Although different types of PHEs may be perceived as very different situations, they all require mental health services to pivot away from normal practice into emergency response mode. This pivot varies geographically, and by the type of PHE, however, many aspects are consistent irrespective of the situation, for example, the initiation of emergency decision-making structures, new staff responsibilities, and increased reliance on alternative service delivery modes such as remote consultation and patient transfer. A substantial body of literature on mental health interventions delivered during public health emergencies as well as variation in research methodologies and application of interventions for different PHEs, provides justification for use of a scoping review design. The objective of this review is to report on and map the existing evidence to gain a clearer understanding of the available mental health interventions and supports, their relevance to the public health emergency scenario, and the characteristics of the population they target. Therefore, the aim of this review will be to explore how have mental health services respond to previous PHEs with a specific focus on the effectiveness of responses, as well as barriers and facilitators to implementation. 

## Methods

The structure of this protocol aligns with the five-stage Arksey O’Malley guidance ^13^ further refined by Westphaln and colleagues ^14^. These refined guidelines will also be implemented during the review process. The Preferred Reporting Items for Systematic Reviews and Meta-Analysis Scoping Review extension (PRISMA-ScR) ^15^ also will be used to report on terminology and fundamental components as the review process progresses. The scoping review protocol will be published in HRB Open.

### Selection of relevant studies

***Eligibility criteria*.** Primary and/or secondary research publications involving individuals with pre-existing or newly developed mental health conditions who engaged with mental health services in instances of PHEs are of interest for this scoping review. Language restrictions will not be imposed in the search and selection process. Regarding methodology all research methods and study designs will be considered for inclusion. Studies exclusively focused on the epidemiological aspect of mental health conditions during PHEs will be omitted, as this review focuses exclusively on the provision of mental health services during emergencies. Please see the summary of the eligibility criteria in Table 1. Below are the detailed eligibility criteria structured within the Population, Concept, Construct (PCC) construct for clarity.

**Table 1.** Inclusion and Exclusion Criteria.

Inclusion Criteria	Exclusion Criteria
Publications related to any mental health interventions/ supports delivered during a public health emergency.	Publications that concentrate on mental health interventions or supports provided prior to or following public health emergencies, e.g., digital mental health and psychosocial support programme before pandemic, Long COVID, prolonged military conflict
Publications related to any reorganisation of mental health care due to PHEs.	Publications that only focus on the epidemiology of mental health conditions during PHEs.
Publications regarding mental health Interventions and/or supports that were provided to populations of any age who had existing or emerging mental health conditions.	Mental health policy documents.
Publications that use any research methodologies and study designs. Primary and/or secondary research publications with objectives such as effectiveness, implementation, evaluation, or description of mental health interventions/supports.	

**Population**

The scope of this review encompasses research about individuals dealing with pre-existing or newly developing mental health conditions and/or suicidal thoughts and behaviours, including but not limited to psychosis, mood disorders, post-traumatic stress disorder (PTSD), depression, anxiety, self-harm suicidal attempt. The focus is on those who have actively engaged with mental health services in the context of PHEs.

**Concept**

Publications that address any modifications to mental health systems, community-based support structures and psychosocial interventions that have been implemented in response to the impact of an emergency will be included. All studies that have documented or investigated the implementation or evaluation of mental health interventions or support provision during PHEs will be included. For the purpose of this review, the concept of mental health intervention and/or support is operationally defined as any adjustment to mental health care introduced at a national, regional, or community level as a consequence of a public health emergency documented in academic or grey literature.

**Context**

The scoping review will include any studies that concentrate on mental health interventions, or the provision of supports delivered across various settings and to diverse populations during public health emergencies, including pandemic, and/or man-made or natural disaster, and/or war, or military conflict.

The research inquiry outlined above was formulated as a direct response to a preliminary exploration undertaken within the Medline database. This initial exploration revealed the substantial volume of publications indicating varying nature of the literature in terms of study designs and types of mental health interventions and supports deployed within distinct PHEs.

***Search strategy*.** The review will involve searches across both academic and grey literature sources. Academic databases to be searched are Medline via Ovid, EMBASE via Ovid, PsycINFO via Ovid, CINAHL via EBSCO, Web of Science, and Cochrane Central Register of Controlled Trials while grey literature searches will involve searching the Social Care Institute for Excellence (SCIE) database, WHO Library and Digital Information Networks database, The DART Europe E-thesis Portal, United Nations iLibrary database, The National Academies Press database extension for emergency preparedness and disaster management, and first fifty results of the Google Scholar database. Additionally, the reference list of identified articles will be reviewed to identify any additional sources that may be relevant for this scoping review. In cases where there is duplication of sources, such as when a primary source is used in evidence synthesis, the primary source will be excluded if context of evidence synthesis is relevant to this review.

The search strategy for this review encompasses a wide range of terms related to mental health conditions, the concept of interventions and supports, as well as terms related to various types of PHEs. Please refer to Table 2 for the list of key terms developed in alignment with the PCC (Population, Concept, Context) construct. The key terms have been sourced from previous research publications, enhancing their relevance. The use of MeSH headings, Boolean operators, truncation, and phrases enclosed in inverted commas will be applied where appropriate to optimise the search query. The University College Cork librarian contributed to the development of a search strategy applicable to different search engines. Considering the broad and evolving nature of mental health interventions delivered during PHEs, the search strategy may be altered if additional terms or sources of evidence are uncovered.

**Table 2.** Key terms.

**Population**	“mental health” OR suicid* OR self-harm OR “self harm” OR selfharm OR “non-suicidal self-injury” OR depress* OR anxiety OR trauma OR “psychological health” OR “post-traumatic stress disorder” OR ptsd OR psychosis OR “mood disorder” OR “substance use disorder”
**Concept**	“psychological response” OR “psychosocial response” OR “psychosocial support” OR “crisis management” OR “crisis intervention*” OR “psychological first aid” OR “face-to-face” OR “tele-mental health” OR “psychosocial intervention”
**Context**	“public health emergenc*” OR pandemic* OR COVID-19 OR “infectious disease*” OR “natural disaster*” OR “extreme weather event*” OR “climate disaster*” OR disaster* OR tsunami OR flood* OR “bush fire*” OR “wild fire*” OR “ice storm*” OR tornado* OR hurricane* OR avalanche* OR drought OR landslide* OR famine OR “nuclear disaster*” OR war* OR “military conflict”

### Charting data

***Data management*.** Rayyan software ^16^ will be used to manage data, including the removal of duplications, screening, and data extraction of identified articles.

**Figure 1.**
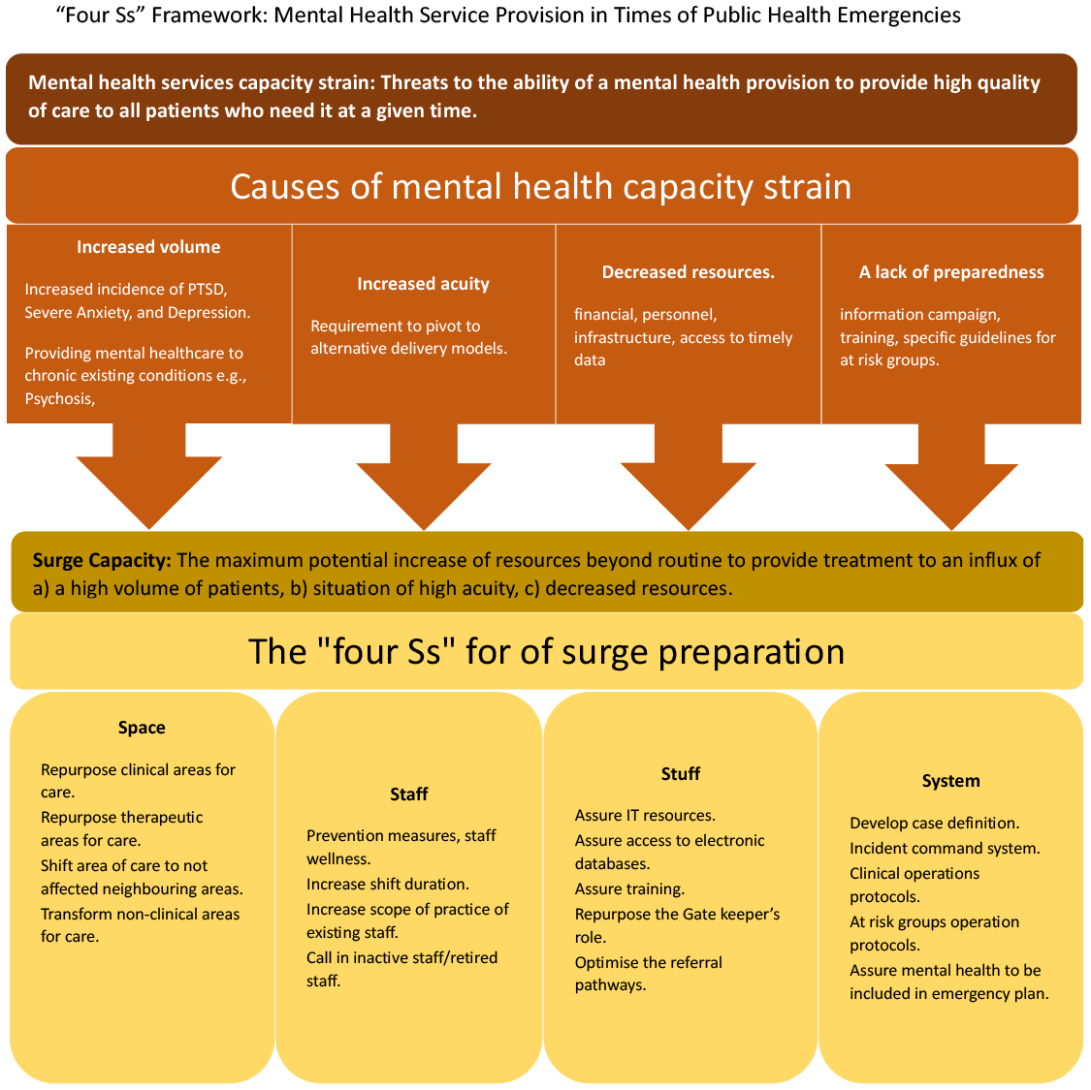
“Four Ss” framework.

Incorporating the framework adapted from the Dr. Anesi and his colleagues, known as “Four Ss” ^17^, will offer a structured and systematic approach to organise and chart data. This comprehensive framework offers four primary categories: Space, Staff, Stuff, and System, each further subdivided into more specific themes, as illustrated in Figure 1. It is important to highlight that any themes that will emerge during the review process and were not originally included in the “Four Ss” framework will either be reported separately or incorporated as an extension of the adaptive “Four Ss” framework. This will allow for a detailed exploration of the evidence and will ensure that all relevant aspects are considered and reported in this review.

***Selection process*.** The initial screening will involve assessing titles and abstracts for eligibility. The subsequent full-text review of publications that pass the initial screening, conducted against inclusion and exclusion criteria, will ensure selection of only most relevant publications. Two reviewers will autonomously evaluate titles, abstracts, and full text of publications. Any disagreements during the review process will be resolved through consensus discussion or recourse to a third reviewer opinion. A pilot screening will be carried out with five publications at the beginning of each stage of the review.

***Data extraction*.** The full-text screening process will determine the eligibility of publications for data extraction. A predefined data extraction tool designed in Excel (XLSX) will be utilised to extract information of interest for this scoping review. These includes lead author, year of publication, study design/methodology, location, population, PHE type, and fields related to the interventions and/or supports delivered during PHEs created in accordance with the adapted "Four Ss" framework. Please see the template of the data extraction tool in Table 3. Importantly, if any new themes develop during the review process, these will be isolated individually or incorporated into the adaptive “Four Ss” framework. To ensure consistency, the data extraction tool will be tested on a small subset of data retrieved independently by two reviewers. Any discrepancies will be discussed and resolved by wider research team prior to applying data extraction to all included publications. One reviewer will extract data from all included publications, while a second reviewer will double-check all collected data.

**Table 3.** Items of data extraction tool.

Items
1. First Author
2. Year of publication
3. Geographical location
4. Study design/methodology
5. Study population a. Sample size b. Age (Mean and Standard Deviation) c. Gender (proportions) d. Mental health condition (proportions)
6. Type of public health emergency
7. Name and or type of Intervention and/or support provided. *Note. The descriptive publications will be reported as a proportion. The effectiveness studies will be reported as a Standardized Mean Difference SMD of specific intervention, where available. The evaluation studies with consideration of barriers and facilitators will be reported in accordance with the “Four Ss” framework in qualitative (thematic analysis) and quantitative (proportion) forms. a. Space i. Clinical areas for care. ii. Therapeutic areas for care. iii. Shift the area of care to not affected neighbouring areas. iv. Shift the area of care to remote delivery of care. v. Transform non-clinical areas for care. b. Staff i. Prevention measures, staff wellness, safety, ii. Maintain mental health staff capacity. iii. Adapt scope of practice of existing staff, training iv. Call in inactive staff/retired staff. c. Stuff i. IT resources. ii. Access to electronic databases. iii. Repurpose the Gate keeper’s role. iv. Optimise the referral pathways. v. Social cohesion vi. Cultural sensitivity vii. Safety, privacy and confidentiality viii. Coping strategies ix. Stigma d. System i. Develop case definition. ii. Availability and accessibility of services iii. Emergency decision-making structures iv. Incident command system. v. Clinical operation protocols. vi. Operation protocols for at risk groups of the population. vii. Mental health to be included in overall emergency plan.

***Data analysis*.** The planned approach for reporting and presenting data will involve various forms to include graphs, tables and narrative description of results to effectively convey the varying nature of mental health interventions and/or supports provided during PHEs. The initial stage of analysis will involve quantitative (proportional) assessment of mental health responses to specific PHEs using the “four Ss” framework. This approach will allow us to recognise the types of interventions and supports available, as well as the determinants associated with these interventions. Next step will involve quantitative estimation of an effectiveness of the interventions in specific PHE context. The review will provide aggregated estimates (Standardised Mean Difference; SMD) to convey the effectiveness of interventions, where possible. Lastly, the review will quantitatively (proportions) and qualitatively (thematic analysis) explore the barriers and facilitators of the implementation of mental health service responses to PHEs. The alignment of all extracted data with the “Four Ss” framework ensures a structured and systematic analysis that will contribute to comprehensive understanding of the evidence in context of space, staff, stuff, and system. This approach to the reporting and analysing data is likely to yield nuanced and valuable insights into the nature of mental health interventions provided during PHEs.

## Deviation from the protocol

Given broad nature of mental health interventions and supports delivered during specific PHEs it is prudent to acknowledge the potential for deviation in the review methodology during the review process. The deviation can arise particularly in the included publications and emergence of new themes. Any such changes will be documented and reported in the final report after completing the review process, in comparison to the original scoping review protocol.

## Data Availability

No data are associated with this article.
