# Supplementary material for: Factors influencing mental health service delivery during public health emergencies: a scoping review protocol
Source: HRB Open Res. 2024 Feb 16;7:5. [Version 1] doi: 10.12688/hrbopenres.13850.1 (PMC11259773; doi:10.12688/hrbopenres.13850.1)
Supplement: Peer review history

# Reviewer report: Reviewer response for version 1

**DOI:** 10.21956/hrbopenres.15176.r40796

**Published:** 2024-07-19

**Authors:** Yashi Gandhi

**Affiliations:** 1 London School of Hygiene and Tropical Medicine, London, UK

The scoping review focusses on understanding an important domain- one that highlights ‘what works’ and ‘what does not work’ when adapting existing mental health service delivery models in the wake of unplanned public health emergencies. The review seems timely, comprehensive and feasible in its methodology. The authors also describe a clear framework and approach for analysis- the combination of deductive and inductive ensures that all data is analysed and reported. The potential lessons that will be synthesised will help guide future work in planning, designing and implementation of appropriate responses to different kinds of emergencies. 

 A few suggestions that the authors may want to consider:

1. It would be helpful to justify the global focus of the review and how this will be accounted for during data analysis and synthesis. Given that countries differ significantly in the resources available to them (e.g. health staff, financing, etc), it may be crucial to demonstrate what is possible in high resource settings v/s in low resource settings and how the implementation of mental health service provision can therefore be contextualised.
2. In addition to backward citation (i.e. looking at references of included studies), the search strategy can be made more comprehensive by also including forward citation (i.e. publications that have cited the included study/ paper).
3. Suggest specifying what criteria will be used by the authors to determine if they will (1) try to come to consensus OR (2) choose to include a third reviewer when there is disagreement about inclusion of a paper. It is preferable that one method is chosen to ensure consistency in decision making. 
4. It may be helpful to clarify how the authors define ‘mental health problems’. While the authors do indicate some examples (a mix of common and severe mental health problems), it may be helpful to indicate how and why they have chosen only specific conditions in the search strategy. For instance, there are a lot of severe mental health problems apart from psychoses- are they not being considered? Similarly, substance use problems can also have additional search terms (e.g. alcohol use disorders) added to make the strategy more comprehensive.
5. While the authors mention that publications that use any research methodology and study design will be considered, it is not clear if other systematic/ scoping/ literature reviews and commentaries/ opinion pieces are also being included? Typically, they are not considered in reviews but it would be good to clarify and justify the rationale of including/ excluding them. 
6. Suggest the authors clearly mention how they will review papers not in English so that others can replicate these methods if/when necessary. For e.g., if they have review members who are able to screen in different languages, if they plan to use translation softwares, etc. 
7. Suggest adding the percentage of the final list of included papers that will be pilot tested using the data extraction template.

Is the study design appropriate for the research question?

Yes

Is the rationale for, and objectives of, the study clearly described?

Yes

Are sufficient details of the methods provided to allow replication by others?

Partly

Are the datasets clearly presented in a useable and accessible format?

Not applicable

Reviewer Expertise:

Global Mental Health, Implementation Science, Primary Health Care

I confirm that I have read this submission and believe that I have an appropriate level of expertise to confirm that it is of an acceptable scientific standard.

# Reviewer report: Reviewer response for version 1

**DOI:** 10.21956/hrbopenres.15176.r38310

**Published:** 2024-03-05

**Authors:** Francine Cournos

**Affiliations:** 1 Columbia University Mailman School of Public Health, New York, NY, USA

The topic is very important and the review methods are clearly defined.

 The literature on this topic has many shortcomings. The most significant problem is that it often does not distinguish between a person having a diagnosed psychiatric disorder, as defined in the DSM and/or the ICD, versus a person having symptoms of distress without meeting criteria for a psychiatric diagnosis. This is important because most evidence for psychiatric treatment is focused of people with disorders and because there is a shortage of behavioral health care providers, which makes good triage very important.

 This relates to another problem with this literature, which is how to prevent psychiatric disorders. It may be that treating sub-threshold symptoms will prevent the development of full-blown mental disorders, but this goal is rarely addressed. Approaches to sub-threshold symptoms could be very diverse and include intervening with the social determinants of health, such as food and shelter. These measures are not, strictly speaking, mental health treatment.

 The authors will improve their contribution to the literature if they address the above issues.

Is the study design appropriate for the research question?

Partly

Is the rationale for, and objectives of, the study clearly described?

Yes

Are sufficient details of the methods provided to allow replication by others?

Yes

Are the datasets clearly presented in a useable and accessible format?

Yes

Reviewer Expertise:

I'm a psychiatrist at an academic medical center who has engaged in diverse administrative, clinical, public service and research activities.

I confirm that I have read this submission and believe that I have an appropriate level of expertise to confirm that it is of an acceptable scientific standard.

# Response

**Published:** 2024-04-11

**Authors:** Pawel Hursztyn

**Affiliations:** National Suicide Research Foundation, Cork, County Cork, Ireland

Thank you for your insightful comments and for highlighting the key shortcomings in the literature.

 Indeed, the lack of distinction between diagnosed psychiatric disorders and symptoms of distress without meeting diagnostic criteria is a significant issue. Focusing predominantly on individuals with diagnosed disorders overlooks those experiencing distressing symptoms but not meeting formal diagnostic criteria. This is particularly relevant given the shortage of behavioural health care providers, emphasising the importance of effective triage.

 In response to this inquiry, I want to assure you that we will thoroughly cover the plans for adapting triage/preventive services in emergencies. I have gathered several examples of these publications, which I believe align closely with our research objectives that will be highlighted by our findings:

- [Application of the triage assessment system for a rapid assessment of mental health of the referral wounded and their family members in Wenchuan earthquake] - PubMed (nih.gov)
- Mental health response to community disasters: a systematic review - PubMed (nih.gov)
- Preventing after-effects of disaster trauma: the information and support centre - PubMed (nih.gov)
- Service provision in disaster preparation, response, and recovery for individuals with predisaster mental illness - PubMed (nih.gov)
- Triage of disaster-related neuropsychiatric casualties - PubMed (nih.gov)
- [Reliability and validity of the self-reporting questionnaire for assessing mental health applied in Wenchuan earthquake] - PubMed (nih.gov)
- Psychiatric issues in medical-surgical disaster casualties: a consultation-liaison approach - PubMed (nih.gov)

Additionally, your point about preventing psychiatric disorders by addressing sub-threshold symptoms is crucial. While treating sub-threshold symptoms may prevent the development of full-blown disorders, this preventive aspect is often overlooked in the literature. I want to highlight a publication that directly addresses the issue of undiagnosed mental health conditions requiring a more public health-oriented approach during emergencies. https://www.cdc.gov/pcd/issues/2020/20_0261.htm Examples from New York City's ThriveNYC: Delivering on Mental Health - PMC (nih.gov) and Philadelphia's Applying the Policy Ecology Framework to Philadelphia’s Behavioral Health Transformation Efforts - PMC (nih.gov) where a public health approach was taken to address the issue of mental health during emergencies, are also included in our scoping review.

 The issue of undiagnosed mental health conditions as well as the triage system during emergencies will definitely be highlighted in our findings.

 We also agree that interventions targeting social determinants of health, such as access to food and shelter, can play a vital role in addressing sub-threshold symptoms and should be considered within the broader context of mental health treatment. However, while broader non-clinical interventions/supports delivered during PHEs such as shelter and food are fundamental for mental health, they are outside the scope of this review. I can assure you that we will acknowledge these aspects in the discussion section of the scoping review.
